# Stereological and histopathological evaluation of the effect of Thymoquinone on peridural fibrosis following laminectomy in rats

**DOI:** 10.3906/sag-2006-147

**Published:** 2021-02-26

**Authors:** Dursun TÜRKÖZ, Aytaç TÜRKÖZ, Mehmet Emin ÖNGER, Enis KURUOĞLU

**Affiliations:** 1 Department of Neurosurgery, Faculty of Medicine, Ondokuz Mayıs University, Samsun Turkey; 2 Department of Histology and Embryology, Faculty of Medicine, Ondokuz Mayıs University, Samsun Turkey

**Keywords:** Laminectomy, peridural fibrosis, thymoquinone

## Abstract

**Background/aim:**

This study’s aim was to investigate the effects of thymoquinone, which is the essential bioactive component of the volatile oil of *Nigella sativa* on the peridural fibrosis in rats following laminectomy.

**Materials and methods:**

Twenty female Wistar Albino rats were used in our study. The rats were randomly divided into 2 groups: Sham and Surgery + Thymoquinone. Both groups underwent laminectomy at L1 under general anesthesia. The Sham group was not subjected to any drug application. The 2nd group was treated with intraperitoneal 10-mg/kg thymoquinone once per day for a period of 28 days, following the same surgical procedure. All of the group specimens were sacrificed after 4 weeks, and the laminectomy area was examined in terms of new bone volume, capillary volume, and fibrosis volume using stereological approaches.

**Results:**

Statistically significant differences were found between the Sham and Surgery + Thymoquinone groups in terms of new bone volume (P = 0.01), capillary volume (P = 0.01), and fibrosis volume (P < 0.001). It was noted that Thymoquinone caused a significant increase in new bone volume, vascular volume and, a significant decrease in fibrosis volume.

**Conclusion:**

The results of our study indicate that thymoquinone is effective in decreasing peridural fibrosis when applied to a laminectomy model.

## 1. Introduction

Annually, a large number of people undergo surgery of the lumbosacral region, making this operation one of the most applied treatments for spinal disorders [1,2]. The failed back syndrome is identified as a persistent pain in the lower back, leg, hip, and thigh in patients who have had the surgical procedure known as a laminectomy. It is also called a postlaminectomy syndrome (PLS) [3]. Inappropriate application of the surgical procedure, the disruption of the internal disc, spinal stenosis, and peridural fibrosis are among the possible causes of failed back syndrome [4,6]. Peridural fibrosis is known as one of the most common causes of failed back syndrome and possesses characteristic features such as the accumulation of scar tissue between the dura mater and surrounding tissues [7]. The formation of the scar tissue causes the nerve roots to become more prone to damage by disc protrusions by restricting their movement. Peridural fibrosis is suggested as one of the primary causes of pain following spinal surgeries [8]. In the literature, there are no established treatments of peridural fibrosis, and some painkiller drugs are utilized for the alleviation of pain [9]. As the granulation tissue is formed following surgery, peridural fibrosis may increase the incidence of surgical complications [10].

Thymoquinone is the bioactive compound of the volatile oil of black cumin (C10H10O2; 2-isopropyl-5-methyl-1, 4-benzoquinone). Thymoquinone is acquired from the seeds of *Nigella sativa* that are bound up in the Ranunculaceae family [11,12]. Thymoquinone has been used for various purposes such as for its antioxidant, antiinflammatory, immunomodulator, anticancer, antimicrobial, antihistaminic, and antineoplastic properties [13–15]. Thymoquinone inhibits the cyclooxygenase (COX) and lipoxygenase (LO) pathways, which are the enzymes that mediate inflammation [16]. Moreover, it was determined that Thymoquinone inhibits the capacity to produce cellular LO, which is synthesized for L-arginine in the presence of the enzyme nitric oxide synthase and is associated with several disorders. Thymoquinone is known to inhibit NO production at a percentage of 95% [17,18].

Several studies about peridural fibrosis can be found in the literature. Some previous research has indicated that antineoplastic agents like mitomycin C, 5-Fluorouracil, and cyclosporin reduce peridural fibrosis, although there are side effects and the cost is high. Furthermore, there are studies focusing on the external particle radiation but no beneficial effects on peridural fibrosis have yet been determined. The solution for the problem of peridural fibrosis is continuously being researched [19–21], but a standard approach and treatment protocol have not been previously established.

In this study, we aimed to investigate the possible effects of intraperitoneal application of Thymoquinone, which is the basic bioactive compound of the *Nigella sativa* volatile oil, on the experimental laminectomy model of rats with histopathological and stereological methods.

## 2. Materials and methods

### 2.1. Experimental animals

This study was performed following the ethical approval of Ondokuz Mayıs University (2014/30), in Ondokuz Mayıs University’s Experimental Animals Research Center. In this experimental study, 20 female Wistar Albino rats, weighting between 280–300 g were used. All of the subjects were kept in rooms with stable temperature and humidity, given normal tap water, and fed rat pellets without any limitation or additional food. The histopathological and stereological evaluations of the samples were performed in the Department of Histology and Embryology laboratories at the Faculty of Medicine of Ondokuz Mayıs University.

Two groups were determined in the experimental design: the first was the Sham group. In this group, 10 rats were subjected to the operational laminectomy procedure at the L1 level. The 2nd group, the Surgery + Thymoquinone group, consisted of 10 rats that were subjected to same laminectomy operation. After the operation, Thymoquinone was applied intraperitoneally for 28 days at a dose of 10 mg/kg/day [22].

### 2.2. Procedure 

For prophylactic purposes, a single dose of 50-mg/kg ceftriaxone (Rocephine, Roche, Turkey) was applied intraperitoneally 30 min before the operation. General anesthesia was established in the rats, which were not given any food 1 night prior to the operation, with an intraperitoneal injection of a mixture of Ketamine (50 mg/kg) (Ketalar, Eczacıbaşı, Turkey) and Xylazine (10 mg/kg) (Rompun 2%, Bayer, Turkey). The anesthesia was regulated to keep the rats unresponsive to painful stimuli without interrupting their spontaneous respiration; additional doses were given when required.

#### 2.2.1. Formation of the laminectomy model

**Figure 1:**
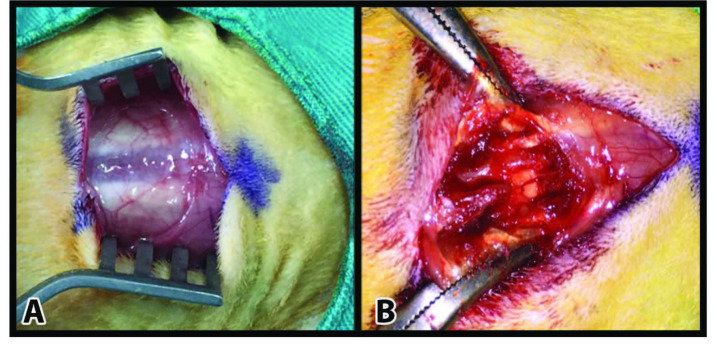
Photo of the paravertebral muscle and fascia (A) and exposure of the spinous process after the incision (B).

The rats were kept stable by having their extremities and their tails set in a prone position on specially manufactured wooden platforms. The surgical areas were then shaved, and disinfection was performed with povidone iodine solution (POVIOD; 10% polyvinylpyrrolidone-iodine complex, Saba, Turkey). The operational area was covered with sterile coverings. A 3-cm skin incision was made on the spinous processes with lumbar 1 vertebra in the middle of the incision, and the paraspinous muscles were then removed with blunt dissection. Small automatic retractors were used to uncover laminas and spinous processes. With the help of a surgical microscope (Leica RM 2135, Leica Instruments, Nussloch, Germany), dura mater was exposed by making a total laminectomy to the L1 vertebra (Figures 1A and 1B). The layers were properly closed in all groups following hemostasis [23].

#### 2.2.2. Intraperitoneal application of thymoquinone:

The surgery day was noted as day 0 and immediately after, intraperitoneal Thymoquinone was applied to Surgery + Thymoquinone group subjects at a dose of 10 mg/kg/day for 28 days.

The Sham and Surgery + Thymoquinone groups were both handled in their own cages. At the end of the 4 weeks, they were sacrificed with intraperitoneal high-dose (75-100 mg/kg) Thiopental sodium (Pentothal Sodium, Abbott, Italy). During the collection of the samples, the animals were checked for dura tear, nerve damage, and infection. There were no spotted dura tear or infections in the rats. The vertebral columns were removed and were put in 10% formaldehyde for histological tissue processing.

### 2.3. Histological analysis

The vertebrae samples obtained from the subjects were kept in 10% formaldehyde solution for postfixation for 1 week and then decalcified in 5% formic acid solution for 21 days. The light microscopical routine histological tissue processing steps were performed for the samples following decalcification. Ten micrometer sections in thickness were taken from every paraffin block in accordance with stereological approaches from the results of the pilot study and stained with Hematoxylin and Eosin. 

**Figure 2:**
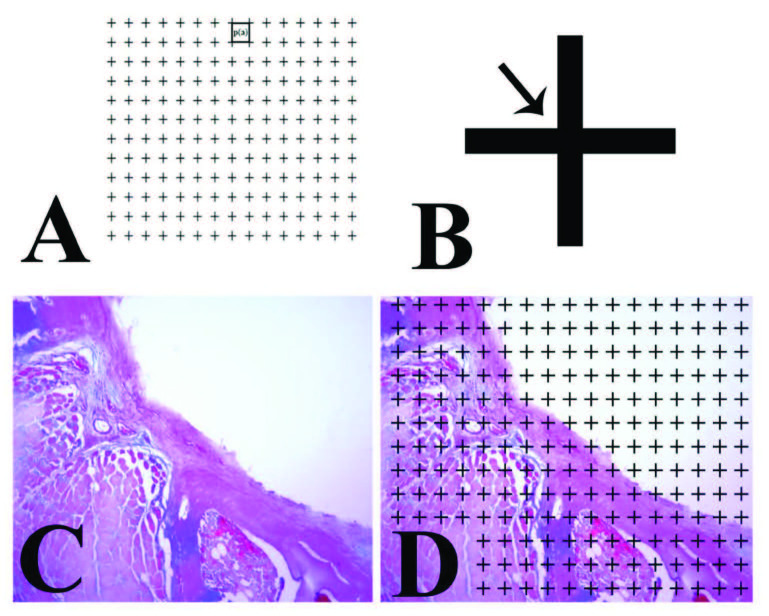
Application of Cavalier’s principle on the sections. Point-counted grid (A) was used to perform the stereology. Every point represents a certain area (A and B) and, after putting the point-counted grid on the section, counting was performed to obtain the total area of the related parameters (C and D).

The section-sampling fraction was determined as 1/4, and the sections were taken in 10-µm thickness. The point density of the grid was determined according to the coefficient of error [24]. The volume estimations of new bone, new capillary, and fibrosis in the laminectomy area were performed according to the Cavalieri method (Figure 2A–2D), using the following formula [25]. 

Area = a/p (µm x µm) × (ΣP) µm2

a/p: area between 2 points 

ΣP: the total number of points intersecting with the area of interest

Volume = t × a/p × ΣP

### 2.4. Statistical analysis

The SPSS 20 (IBM Corp., Armonk, NY, USA) program was used for the statistical evaluation of the data. Student’s t-test was used for comparing the groups. P values less than 0.05 were accepted as statistically significant.

## 3. Results

**Figure 3:**
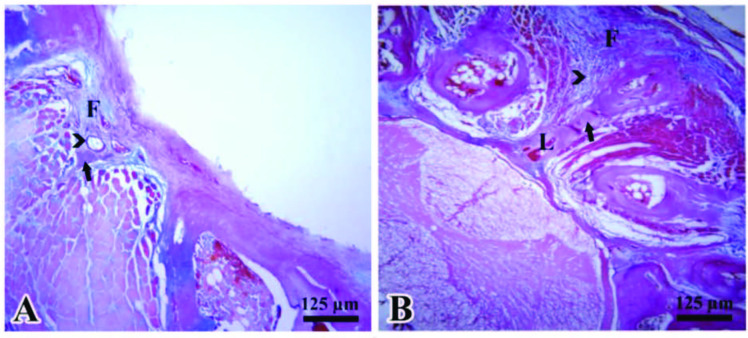
Representative histological images show the laminectomy areas in the Sham (A) and Surgery + Thymoquinone (B) groups, respectively (Arrowheads: Capillaries exist in the fibrosis area (F); Black arrow: New bone; L: Lamina).

The new bone volumes of the Sham and Surgery + Thymoquinone groups were found to be 1.495 ± 0.075 (mean ± SEM) µm3 and 2.265 ± 0.133 µm3, respectively. There was significant difference between the groups (P = 0.01) (Figure 3A, 3B).

Regarding new capillary volume, it was found to be 0.780 ± 0.131 µm3 in the Sham group and 1.897 ± 0.09 µm3 in the Surgery + Thymoquinone group, respectively. There was statistically significant difference between the groups (P = 0.01) (Figure 3A, 3B).

The mean fibrosis volume was found to be 3.151 ± 0.147 µm3 in the Sham group and 1.936 ± 0.095 µm3 in the Surgery + Thymoquinone group, respectively. Similar to the previous parameters, there was also significant statistical difference between the groups (P < 0.001) (Figure 3A, 3B).

## 3. Discussion

Lumbar pain is one of the most common medical complaints and the second most common reason for seeking professional medical help [26]. It is estimated that approximately 200,000–350,000 patients in the US undergo lumbar surgery due to reasons such as discogenic back pain, trauma, tumor, spondylolisthesis, and spinal stenosis [20]. In the laminectomy area, the fibroblast population increases due to activation of the inflammatory cytokines. Growth factors for the repair of local defective vertebral area and large amounts of collagen fibers are formed. With the occurrence of collagen fibers, fibroblasts become fibrocytes. Scar tissue is formed from fibrous connective tissue [27]. Compression of the spinal cord due to peridural fibrosis may lead to the occurrence of unwanted clinical results following surgery [4,28]. The scar tissue that might follow the surgical operation can cause constriction of the nerve root and stenosis [29]. Although it is not completely clear how clinical symptoms and adhesion formations are related, peridural fibrosis is associated with many cases of failed back surgery [28]. Besides the radicular symptoms due to nerve constriction, scar formation may also cause increased durotomy during revision surgeries [30]. The process of fibrosis is regulated by myofibroblast cells as they produce collagen fibers [31]. The exact mechanism behind the formation of peridural fibrosis after surgery has not yet been clearly explored. A previous study reported that the laminectomy membrane is formed due to the invasion of fibroblasts from spinal muscles [32]. Peridural adhesion following spinal surgery might lead to the secretion of vasoactive factors and inflammation, which consequently result in collagen formation and the infiltration of fibroblasts and macrophages.

Thymoquinone, which is the basic component of the volatile oil of *Nigella sativa,* has the formula (C10H10O2; 2-isopropyl-5-methyl-1,4-benzoquinone) and has been used for years as an antioxidant, antiinflammatory, and antineoplastic agent [13,14]. In their study, Awad et al. stated that thymoquinone is promising agent for defense against fibrosis, hepatic steatosis, oxidative stress, and inflammatory apoptosis [33]. Moreover, it was also previously suggested that thymoquinone is a scavenger of free radicals and superoxide radicals and that it protects the functionality of various antioxidant enzymes [34]. In their study, Pourgholamhossein et al. demonstrated that thymoquinone is protective against lung fibrosis as it displays features of oxidative stress inhibition and profibrotic gene down regulation [35]. In addition, thymoquinone is currently being used for cancer treatment [36]. Ahmad et al. found that it has the potential to be a chemopreventive agent in cancer studies and use in antitumor therapeutic paradigms [37].

Thymoquinone is used in the management of inflammatory and autoimmune diseases as it inhibits NO production approximately at 95% [17]. Also, thymoquinone has an inhibition effect on COX and LO pathways in the arachidonic acid metabolisms of rat peritoneal leucocytes [38]. Besides, in an allergic airway inflammation model in rats, thymoquinone caused inhibition of PGD2 and COX-2, resulting in an antiinflammatory effect [39]. 

In the current study, the aim was to assess the effect of thymoquinone application following laminectomy on new bone formation. When the results were evaluated, it was observed that thymoquinone application causes significant increase in new bone volume and vascular volume (P = 0.01), whereas it caused a significant decrease in fibrosis volume (P < 0.001). These results are in agreement with the results of the previous study of Wirries et al., in which they suggested that Thymoquinone may induce osteogenesis [40]. In a previous study, Kirui et al. showed that sustained thymoquinone application increases bone healing [41]. Also, there are several studies that demonstrated that thymoquinone application has protective and beneficial features against spinal cord ischemia/reperfusion injury as it decreased the concentration of oxidative products, as well as the reduction of neuropathic pain in parallel to our results [42,43].

Masson’s Trichrome staining is one of the best ways to show peridural fibrosis. However, in this study, we showed quantitative evaluation of peridural fibrosis stereologically; therefore, we prefered H & E staining as this staining method is feasible for stereological estimation.

Although further physiological, biochemical, and electron microscopical examinations, such as investigating hydroxyproline levels are required, our study may guide future studies and contribute to the scientific literature. It is also important to compare the effect of Thymoquinone with a standard drug or investigate the local use of thymoquinone as a different group in future studies. We are of the opinion that the presented results can enlighten the way for more researchers studying this area.

## Informed Consent

Ethical approval was obtained from the Ondokuz Mayıs University’s Animal Studies Local Ethics Committee with number 2014-30.
